# Potential role of proprietary patent medicine vendors in primary eye and ear care: A mixed methods study in northern Nigeria

**DOI:** 10.1371/journal.pone.0326132

**Published:** 2025-06-24

**Authors:** Joseph Okeibunor, Andrew Smith, Emma Jolley, Elizabeth Elhassan, Oluwatosin Adekeye, Lawal Hasfat Kontagora, Sunday Isiyaku, William Adamani, Caleb Mpyet, Hannah Faal, Elena Schmidt, Clare E. Gilbert

**Affiliations:** 1 Social Scientist, Department of Anthropology and Sociology, University of Nigeria Nssuka, Enugu State, Nigeria; 2 Honorary Professor, International Centre for Evidence in Disability, Department of Population Health, London School of Hygiene and Tropical Medicine, London, United Kingdom; 3 Head of Portfolio, Health and Disability Research, Sightsavers, Haywards Heath, West Sussex; 4 Filant Consulting, 1a Yusuf Lere Drive, U/Rimi, Kaduna, Kaduna State, Nigeria; 5 Social Scientist, Department of Psychiatry, Ahmadu Bello University Teaching Hospital, Shika, Zaria, Nigeria; 6 Director, Academic Services, National Teachers’ Institute, Kaduna, Nigeria; 7 Country Director, Sightsavers, Kaduna, Nigeria; 8 Research Officer, Sightsavers, Kaduna, Nigeria; 9 Department of Ophthalmology, University of Jos, Jos, Nigeria; 10 Director of Evidence, Research and Innovations, Sightsavers, Haywards Heath, West Sussex; 11 Adjunct Professor, International Eye Health, University of Calabar Teaching Hospital, Calabar, Nigeria; 12 International Centre for Eye Health, Department of Clinical Research, London School of Hygiene and Tropical Medicine, London, United Kingdom.; University of Turin, ITALY

## Abstract

**Background:**

In Nigeria, patent and proprietary medicine vendors (PPMVs) are permitted to sell a limited range of medication. They are important providers of health care despite their limitations and may be trained to manage specific conditions, such as malaria, but not ear and eye conditions. In this study, PPMV’s knowledge and management of ear and eye problems and community members health seeking behaviour were explored, as well as whether community members would access PPMVs if they were trained in primary ear and eye care.

**Methods:**

Quantitative and qualitative methods were used in a cross-sectional observation study: a survey of 1,591 adults in 40 clusters in two urban and two rural areas; 64 focus group discussions with community members and four with health professionals in two ear, nose, throat and eye clinics; in-depth interviews with ten community leaders, 11 primary health care workers, and 21 hospital staff. A check list was used to assess 36 PPMVs’ facilities and structured questionnaires were administered to 36 PPMVs and 401 hospital patients in ear and eye outpatient departments.

**Results:**

Community members reported that eye and ear problems were frequent but less common than other conditions. Health seeking behaviour was influenced by accessibility, availability, cost of medication, and trust in the provider. Most PPMVs had no formal training, had little knowledge of ear and eye conditions and were enthusiastic about being trained to manage them. Living far (>5km) from a health facility, being male, uneducated and poor were significantly associated with willingness to access PPMV after training in primary ear or eye care.

**Conclusions:**

PPMVs might be able to play a role in delivering primary ear and eye health care for common conditions, in collaboration with local clinicians. To do this, PPMVs would require training in eye and ear conditions and skills in their detection and counselling clients as well as reliable supply chains for medication, and skills in stock control, record keeping and facility management.

## Introduction

Vision and hearing impairments are common, affecting 1.1 billion [1] and 1.5 billion [2] people, respectively. The prevalence of both impairments increase with age, but globally 70.2 million [1] children are vision impaired and 34 million [2] are hearing impaired. Over two thirds of those affected live in low- and middle-income countries (LMICs) [1,2]. Estimates for sub-Saharan Africa (SSA) indicate that approximately 5.3% of the population are vision impaired and 3.6% are hearing impaired, with significant variation by geography and population sub-group [1,2]. The commonest causes of both impairments are amenable to highly cost effective interventions, such as hearing aids for those with age related decline in hearing, topical treatment of infection of the external ear and topical and sometimes systemic treatment of infection of the middle ear, and surgery for cataract and spectacles for refractive errors for distance and near vision impairment [1,2].

In addition to conditions which lead to impairment, other conditions can cause troublesome symptoms. These include earwax, foreign bodies in the ear and middle ear disease, and conjunctivitis, eyelid infections and dry eyes. Data on the prevalence and magnitude of these less severe conditions are not robust, but up to 10% of the population may be affected by non-visually impairing eye conditions at any one time [3,4], and middle ear diseases in children [5] and external eye disease in adults [6] are common, particularly in poor communities.

One of the main reasons why the prevalence of vision and hearing impairment are high in low-income countries is because access to ear and eye care services is poor as they are primarily located in urban and peri-urban areas whereas most of the population live in rural areas [7]. In the absence of easily accessible formal services, affected individuals often resort to traditional remedies, such as couching for cataracts, which can cause harm [8], or a worsening of the condition due to delay in seeking appropriate care. In Nigeria ear and eye care services are mainly provided in secondary and tertiary level facilities in urban areas, leaving rural populations underserved, as primary care does not provide these services in most of the country.

In Nigeria, as in many other countries, there is a wide range of formal and informal providers of health care. Informal providers in the community in many African countries include traditional healers, who have a wide range of practices, such as herbalism, faith healing (diviners), bone setting and attending births [9]. Some provide surgical interventions, such as circumcision and couching (a procedure for cataract) [10]. Other informal providers sell medication; in Nigeria these individuals are called patent and proprietary medicine vendors/dealers (PPMVs) [11]. These informal providers are ubiquitous and numerous, and because they come from and live within the community, they are readily accessible which is often not the case for formal providers.

In Nigeria, private drug retailers include community pharmacists (CPs) and PPMVs are important sources of basic health care delivery [11]. Community pharmacists are trained in pharmacology and/or dispensing and can stock and sell a wide range of medications, including prescription drugs, in registered private outlets under the auspices of their Association. In 2016 there were 12,800 active registered pharmacists for a population of 186 million, approximately 40% of whom worked in the southwest region of the country. PPMVs on the other hand have usually received no formal training and are only permitted to sell drugs approved by the Pharmacy Council of Nigeria. However, the approved list of patent medicines (2012) does not include medication for eye or ear conditions [12]. PPMVs work at village level and virtually every village in Nigeria has an outlet. Indeed, in 2016 there were estimated to be at least two PPMV outlets for every 10,000 population, with a higher density in the south than in the north of the country [13].

Given the ubiquity and accessibility of PPMVs there have been several initiatives in Nigeria and other SSA countries to engage PPMVs in the control of common diseases such as fever, diarrhoea and malaria [11]. In Nigeria, PPMVs are being trained to provide community-based management of uncomplicated malaria, as part of the national strategy for malaria control [14]. National surveys in Nigeria show that PPMVs are the first point of contact for 8% to 55% of illnesses in young children [11] and for 35% to 55% of adult malaria cases who seek treatment [11]. However, studies which address whether people with ear or eye complaints access PPMVs [4,15], and PPMVs’ capacities to manage them are scarce.

The purpose of this study was to explore whether PPMVs could contribute to primary ear and eye care, particularly in under-served areas. The objectives were a) to explore community’s and patient’s health seeking behaviour when they experience problems with their ears or eyes, b) to ascertain what PPMVs know and do about ear and eye conditions and the resources they have to manage them, c) to seek the views of primary health workers and formal ear and eye care providers on the services provided by PPMVs, and d) to explore formal ear and eye care providers views on whether PPMVs could manage these conditions after training.

Nigeria has 36 States and one Federal Capital Territory which are subdivided into 774 Local Government Areas (LGA, equivalent to districts). Health care planning and management has been devolved to State level, but services for eye and ear care are generally lacking. For example, in 2015 there were 140 practising ear, nose and throat (ENT) specialists [16] and approximately 700 ophthalmologists [17], which are both inadequate for a country of over 180 million people.

The projected population of the northern state of Kaduna where the study was undertaken was 6,113,503 in 2012 (from the 2006 census). Most of the population are subsistence farmers, nomadic pastoralists or traders. At the time of the study there were only eight ear, nose and throat (ENT) surgeons and no qualified audiologists, although five audiometric technicians and special education graduates provide audiometric services. There were no local epidemiological data on the prevalence and causes or vision of hearing impairment. There are no recent data on the number of eye care personnel in Kaduna State but the Strategic VISION 2020 Plan for Nigeria 2007–2011 suggested that the North West geopolitical zone, where Kaduna state is located, had a full complement of ophthalmologists, but a deficit of 137 ophthalmic nurses, 41 optometrists and nearly 9,000 primary eye care workers (unpublished document).

The purpose of this mixed methods study was to explore whether PPMVs could contribute to primary ear and eye care, particularly in under-served areas of Nigeria.

## Methods

### Study setting

The study was undertaken in four rural and urban LGAs in Kaduna State, northern Nigeria. Fieldwork took place in November and December 2013. Kaduna State was selected as there are hospitals with ear and eye departments for referrals and for recruiting study participants. The study population consisted of community members, community leaders, primary health care staff and PPMVs. Hospital outpatients with eye and ear conditions, and providers of ENT and ophthalmic services in government facilities were from two ENT and two ophthalmic departments (ENT: National Ear Care Centre, Kaduna, and Gambo Sawaba General Hospital, Sabon Gari. Eye care: National Eye Care Centre, Kaduna, and Eye unit of Barau Dikko State General Hospital).

### Health seeking behaviour of community members and patients

#### Household quantitative survey.

A community-based cross-sectional survey was undertaken by trained field workers, selecting rural and urban communities using multi-stage cluster randomized sampling. Four LGAs were selected, to balance representativeness and practical and logistical considerations. Firstly, all LGAs in Kaduna state were stratified into principally urban and principally rural. Two LGAs were randomly selected from each stratum giving two rural (Kudan and Sanga) and two urban (Kaduna North and Sabon Gari) LGAs with a total population of almost 950,000 (2006 census). Communities in each selected LGA were then listed. In the two rural LGAs, the headquarter towns were excluded. Ten rural communities were then randomly selected in each LGA. In the two urban LGAs, a similar process was adopted, after excluding rural communities. Study households were selected in ten locations in each LGA using the random walk approach, where the central location in each community was identified as a starting point and two data collectors moved in opposite directions and turned right at any junction until the desired number of participants was attained. If the target number was not reached, an adjacent, unselected community was included.

*Sample size calculation:* Using a 50% assumed rate of awareness of health services and a confidence interval of 95% with an estimated 2.5% error margin, a sample size of 1537 ± 38 was calculated. The sample size was rounded up to 1600 households to allow for non-response. Approximately 40 household members were interviewed in each of the 40 communities across the four LGAs. In each household one person aged 18 years and above was interviewed using a structured pre-tested questionnaire. To ensure equal participation by sex, a male and then a female were sequentially interviewed; if more than one person of the required sex was eligible, one was randomly selected.

*Data collection:* Structured interviews were administered by experienced interviewers in Hausa or English according to the preference of participants. Data were collected on socio-economic characteristics of individuals (e.g., level of education) and households (e.g., assets ownership, access to water, sanitation and electricity, and general health conditions (21 items), based on questionnaires used in similar communities. Questions on eye and ear or vision and hearing problems (3 items), health seeking behaviour in general and for eye and ear care (7 items), and perceptions of ear and eye problems (21 items) were drafted, agreed by the Steering Committee and then pre-tested. Data collection took place over two months at the end of 2013.

*Data management and analysis:* Quantitative data were entered into a database created in EPI Info version 6.04 and analysed using the Statistical Product and Service Solutions (SPSS) version 19 and STATA version 15. Wealth quintile scores were derived using multiple correspondence analysis. Univariate associations were examined using chi-squared tests and variables found to have a p-value of 0.02 or less were added to a multivariate model.

#### Focus group discussions with community members.

In each study cluster one or two separate focus group discussions (FGDs) were held with younger and older men, and younger and older women with up to ten participants in each group. Village elders identified community members to take part. Adult males were identified in one community and adult females in the next. The same applied to young males and females. The FGD topic guides explored participants’ views on ear and eye conditions and hearing and vision problems and how they compared in importance with other health conditions; community member’s health seeking behaviour, and satisfaction with the services available and the care provided by PPMVs. A total of 64 FGDs comprising 463 community members were conducted in the 40 communities. All the FGDs and interviews were conducted by trained interviewers and were audio-recorded, after gaining informed consent to record the interview and to use anonymous quotes.

*In-depth interviews with community leaders*: Ten community leaders/ chiefs were invited to participate in individual in-depth interviews. Community leaders were purposively selected considering the availability of PPMVs in their community while ensuring geographic variation based on the location of the community. The topic guides covered the same topics as in the FGDs outlined above.

*Patient survey in health facilities:* A total of 401 consecutive outpatients in the four hospitals were interviewed using structured questionnaires. Patients were asked when their symptoms started and whether they had sought advice or treatment before attending the hospital. Those who gave a positive reply were asked to describe who they had seen and what had influenced their decision, and the advice they had received. If the patient sought advice from more than one source, the same questions were asked for each consultation. Data were entered into Epi-Info and analysed using simple descriptive analysis.

### Resources, activities and perspectives of PPMVs

#### Structured questionnaire and check list.

Thirty-six communities had a PPMV outlet and all 36 PPMVs completed a short questionnaire about their training, qualifications and the services they provide. All 36 PPMV outlets were visited, and a check list was used to assess space and privacy for consultations, whether the outlet had electricity and a refrigerator, the availability of medications to treat eye and ear conditions, and the presence of a license to practice, a register and referral slips.

#### Focus group discussions.

Eighteen PPMVs from neighboring communities were purposively selected and brought together for four FGDs. Recruitment was facilitated by community leaders through the Association who had a list of all registered PPMV members in the community. The topic guide explored PPMVs’ views on the regulations governing their activities, their education and drug dispensing patterns, how they sourced medication and their pricing structure, and whether they received payment in kind. Specific questions were asked about ear and eye and vision and hearing problems and how they manage them, and they were asked about their interest in being trained to address them. The PPMVs were also shown four images of eye conditions with a very short history. They were asked what they thought the problem was and how they would manage the condition.

### Perspectives of health care providers

#### Primary health care workers.

Staff working in primary health care facilities in the study communities were purposively selected for in-depth interviews, with the assistance of community leaders who preferentially selected staff who had worked in the locality for at least two years. The topic guides covered the type, frequency and relative importance of ear and eye conditions, and their perspectives on community members’ health seeking behavior.

#### Ear and eye care health professionals.

Four focus group discussions were also held, two with seven ear care professionals (ENT specialists, nurses, an audiologist and medical assistants), and two FGDs with nine eye care professionals (ophthalmologists, ophthalmic nurses and medical officers). The health professionals were interviewed in their respective facilities. The topic guide asked about common eye and ear conditions and community members’ health seeking behaviour, their views on PPMVs and patients’ experiences of consulting them, and whether they would be willing to train PPMVs in primary eye or ear care.

### Analysis of qualitative data

All qualitative interviews were recorded and transcribed using MSWord and converted into American Standard Code for Information Interchange Rich Text Format (RTF) files. Interviews in Hausa were first transcribed and then translated into English. The transcripts were coded and analysed for emerging themes using Atlas.ti version 6.

### Ethics

Ethical approval was obtained from the ethics committees of the London School of Hygiene & Tropical Medicine, the University of Nigeria Teaching Hospital, Enugu, Nigeria and the Jos University Teaching Hospital, Jos, Nigeria. The study adhered to the guidelines of the Declaration of Helsinki. On the advice of the Steering Committee, verbal consent was obtained from community members and PPMVs which was witnessed and documented by researchers. Written informed consent was obtained from all other participants. All participants were above the age of legal consent. Community participants identified with ear/eye or hearing/vision problems were referred to the nearest relevant health facility.

### Local Steering Committee

A local Steering Committee was convened, comprising representatives from the PPMV Association and the regulatory body of Community Pharmacists; academics experienced in qualitative and quantitative research; public health physicians experienced in working with PPMVs and staff from Sightsavers Nigeria Country Office who provided technical, logistical and administrative support. Regular meetings were held before, during and after data collection.

### Inclusivity in global research

Additional information regarding the ethical, cultural, and scientific considerations specific to inclusivity in global research is included in the Supporting Information (SX Checklist).

## Results

The number of participants and events are summarized in Table 1.

**Table 1:** Number of participants and events.

Method	Participants	Events
Community survey	1,591	40 households in 40 communities
FGDs with community members; separate groups for males/females; youth/adults; urban/rural community members	463	64 groups in 40 communities
In-depth interviews with community leaders	10	2-3 in each LGA
Structured interviews with patients	200 eye patients 201 ear patients	In two facilities In two facilities
FGDs with health professionals	9 eye care 7 ear care	Two FGDs Two FGDs
In-depth interviews with primary health care workers	11	2-3 in each LGA
Structured interviews with PPMVs	36	
Observation of PPMV outlets	36	
FGDs with PPMVs	18	4 groups

In the survey 1,591 individuals were recruited with an equal representation of urban and rural residents and males and females. The median age of survey participants was 40 years; two thirds were Muslim and one third Christian (Table 2).

**Table 2:** Characteristics of community members who took part in the survey.

Variable		n	Percent
Age group (years)	18-29	284	17.9
	30-49	785	49.3
	50-69	360	22.6
	70+	162	10.2
Sex	Male	793	49.8
	Female	798	50.2
Locality	Urban	803	50.5
	Rural	788	49.5
Education attained	None	302	19.0
	Any	1,289	81.0
Income generating activities	None	246	15.5
	Any	1,345	84.5
Marital status	Single	89	5.6
	Married	1389	87.9
	Widowed/divorced/separated	104	6.5
Religious affiliation	Muslim	1068	67.1
	Christian	519	32.6
	Traditional/none	4	0.3
Length of stay in community	0-5 years	144	9.1
	6-20 years	461	29.0
	>20 years	986	62.0
**Total**		**1,591**	**100%**

### Frequency and impact of ear and eye problems

The most common health problems reported by community members were malaria (80.9%), fever (55.6%) and eye/vision problems (38.4%). Ear/hearing problems (28.0%) were the fourth commonest. Focus group participants corroborated the survey findings, saying that although ear and eye problems were frequent, they were not as common as other conditions. They thought that eye conditions tended to affect the elderly whereas ear conditions affected younger people. Community members highlighted that these conditions could impact economic productivity, social status, independence, and self-esteem, and can affect the marriage prospects of women. Eye conditions were attributed primarily to dust, a dirty environment, wind (the Hamarttan) smoke from cooking and occupations such as carpentry, welding, and farming. The most common ear problem was discharge from the ears. Ear problems were attributed to dry wind, flies, hereditary factors and harmful self-treatment practices.

The majority of survey participants reported to have very good or good eye and ear health at the time of the survey (71.4% and 82.2% respectively) and only 4.9% and 3.1% reported poor or very poor eye or ear health, respectively.

### Health seeking behaviour of community members and hospital outpatients

#### Community members.

When asked whom community members would consult with their general health problems, government health facilities were mentioned the most frequently (90.0%)(Table 3). PPMVs were the second most frequent source of advice (46.3%), followed by private hospitals/clinics (22.6%) and traditional healers (18.5%). The pattern was similar for eye and ear conditions. For all three groups of conditions, those living in urban areas mentioned private clinics and PPMV outlets more frequently than those living in rural areas, and rural participants mentioned traditional healers more frequently for all groups of conditions.

**Table 3:** Who community members would consult with their general health or eye and ear complaints.

Health provider	General health		Eyes		Ears	
	N	%^*^	N	%^*^	N	%^*^
Government health centre	1399	90.0%	1342	84.9%	1250	79.1%
PPMV outlet^**^	720	46.3%	497	31.4%	489	30.9%
Private clinics/hospital	351	17.8%	282	17.8%	256	16.2%
Traditional healers^***^	288	18.5%	214	13.4%	212	13.4%
Other^***^	6	0.4%	6	0.4%	6	0.4%
Do not know	24	1.5%	99	6.3%	200	12.7%

* more than one response was possible.

** The terms drug seller/chemist were used in the data collection tools, as community members would not be able to distinguish between different types of sellers of medication.

*** includes spiritual healers and couchers (a traditional procedure for cataract).

When asked about the services provided in health facilities and by PPMVs, a number of differences emerged. Staff in health facilities were considered able to diagnose conditions, dispense drugs, prescribe treatment and counsel patients, whereas PPMVs focused primarily on dispensing drugs and only 2.6% of respondents reported that PPMVs diagnosed conditions. However, if clients do not have a prescription, several community members stated that the PPMVs dispensed the drug clients requested and at doses they could afford. One participant said that PPMVs do not provide any counselling.

*They don’t counsel or give us any health education. They are there to sell their drugs of any kind you ask for...* FGD Adult(A)/Male(M)/Rural(R)/Sanga.

When asked about where they go to receive care for ear and eye conditions, over 90% of respondents named government health centres and only 1–2% mentioned PPMVs.

#### Hospital outpatients.

Two thirds of the 201 ENT patients interviewed lived in urban areas (69%), 54% were female and the mean age was 18 years (range 1–80). More than one symptom was often reported and the commonest were discharge from the ear (42.8%), painful gradual loss of hearing (28.4%), and ringing noises in the ear or head (27.4%). The majority had experienced symptoms for less than 3 weeks (97%). The most common diagnoses were otitis media and otitis externa followed by deafness/presbyacusis and tinnitus. Among the 200 ophthalmic patients interviewed, 68% also lived in an urban area, 57% were female, and the mean age was 33 years (range 1–85). More than half had itchy, watery eyes or red, discharging eye(s), a quarter (25.5%) had experienced gradual painless loss of vision and another 30.5% had difficulty with near vision. The majority had experienced symptoms for less than 3 weeks (95%). The most frequent diagnoses were conjunctivitis in almost half, refractive error and cataract.

Over half of the patients had not consulted anyone before attending the outpatient clinics (57%)(Table 4). One in six eye patients had consulted an optometrist (16%) and one in four ear patients (27%) had visited a health facility or a community pharmacist. Only three patients overall (1%) had consulted a PPMV. Thirty-four had consulted a second provider before attending the outpatient department and four had consulted a third provider, but none involved consultating a PPMV.

**Table 4:** Health seeking behaviour by patients with ear and eye conditions before attending outpatient clinics.

Who was consulted first	Eyes		Ears		Total	
Did not consult anyone	117	59%	111	55%	228	57%
Family member	23	12%	24	12%	47	12%
Health facility	17	9%	29	14%	46	11%
Drug seller in town*	0	0%	27	13%	27	7%
Optometrist	31	16%	NA	NA	34	8%
Audiologist	NA	NA	3	1%		
Community member	9	5%	3	1%	12	3%
Drug seller in village (PPMV)^*^	2	1%	1	0%	3	1%
Other	1	1%	3	1%	4	1%
**Total**	**200**	**100%**	**201**	**100%**	**401**	

* These terms were used, as community members would not be able to distinguish between different types of sellers of medication.

The main factors which influenced the choice of provider made by ENT patients were convenience (73.3%) and cost (15.6%) whereas the two most important factors for ophthalmic patients were convenience (49.4%) and they had faith in the skills of the practitioner (49.4%).

Qualitative interviews corroborated many of the community survey findings and the data provided by patients. However, different groups had different observations of community health seeking behaviour in general and for ear and eye care in particular. The majority of community members said that they preferred to go to a health facility first and would go to a PPMV only to buy medication if the hospital was out of stock, or the medicines were cheaper. Younger participants more likely to visit health facilities, as the availability of medicines in drug stores in their view was unpredictable, and some were concerned by the sale of out-of-date drugs. These participants resorted to PPMVs only if they could not afford hospital charges for medication.

*“We in this community are not willing to accept the services of the PPMVs...as we all rely mostly on going to the general hospital”* FGD Adult(A)/Female(F)/Rural(R), Sabon Gari

*“I prefer to visit the hospital because chemist prescriptions depend on luck”.* FGD, Young(Y)/Female (F)/Urban(U), Kaduna

Many community members described the complex factors involved in the decision-making process, as described by a rural female resident:

*“To me, it is because of no other options do I patronize the PPMVs before going to the hospital, as they are the ones readily available to us due to affordability and accessibility. The PPMVs do not give quality of service, that we know, but what can we do, since to access facilities is not easy?” A/F/R Sanga*

However, a number of respondents said that they would use home remedies or go to a PPMV first and would only go to a health facility if the treatment did not alleviate their symptoms. Home remedies were particularly common when treating children. These statements were corroborated during in-depth interviews with primary health workers (PHW).

*“The parents will …use salt and water solution to give the child relief …”* (FGD, Y/M/U Kaduna)

*“When the symptom [of an ear problem] is noticed, first aid treatment commences immediately, and if it persists, you go to the PPMVs and if it persists, then you go to the hospital for further attention.”* (FGD, A/F/R/ Sanga)

*“About the ears, the first thing some of them do is to use paracetamol and groundnut oil as the first treatment measure, then if no improvement they visit the traditional healer, PPMVs or any nearest health facilities close to them.”* (structured interview (IDI, PHW)

Some *c*ommunity leaders (CLs) were of the opinion that traditional healers were a preferred port of call but there was no consistency across communities.

*“In my community, if you have ten people, seven of them will visit first the local traditional healers for their medication, before going to the PPMVs or the hospital”. (IDI,* CL, Sabon Gari)

Primary health workers and hospital health professionals reported that typical behaviour they observed was self-treatment, followed by treatment from traditional healers or PPMVs, and lastly a health facility. The majority did not approve of the treatment provided by PPMVs and other informal care providers, as they saw the consequences of delayed or inappropriate treatment. They believed that community members accessed these services because they were either uneducated about potential complications or because they were poor and could not afford formal health care services.

*“Due to poverty and ignorance people tend to look for easily and affordable medication within their neighbourhood.”* (FDG, HP eyes)

*“They go to the chemist because of ignorance and poverty thinking they can handle their cases effectively, but they do more harm than good.”* (IDI, PHW, Angwan Shami)

The FGDs with community members described a broad range of factors which influenced communities’ decisions on whether to seek care and from where. The severity of the symptoms influenced when and where care was sought.

*“The stage at which people go to seek medical attention varies depending on the severity of the pain.”* (FGD, A/F/U, Kaduna)

*“The severity of the ear problem as well also affects decision making either to go to the PPMVs, traditional healer, or the hospital*.” (FGD A/F/R Sanga)

Many participants pointed out that the availability of money was a key factor influencing their choice. People who could not afford hospitals opted for cheaper sources of care, usually drug stores.

*“Officials of hospital would say “as soon as you feel any ailment rush to the hospital” but if you get there with little money, they chase you away saying “if you don’t have money why come to the hospital?””* (FGD, Y/F/U, Kaduna)

In many cases, the choice of the health provider was determined by the household head, usually the husband or the parent:

*“The mother may initiate the decision, but the father may complain of money and so may decide that the child be taken to the chemist. If there is no result, later they can go to hospital.”* (FGD, A/F/U Kaduna)

*“Some cannot take decisions by themselves - they have to rely on their husbands or parents, and this causes a lot of consequences as they treat themselves or visit the herbalists.”* (FGD, HP eyes, Kaduna)

Some of the reasons why communities may prefer to visit PPMVs included their proximity, flexibility with payment and opening times, and the perception that they were more caring and responsive than hospital staff.

*“The PPMVs are trying very well for us as they give drugs to relieve the pain, … especially when the incident happens in the night.”* (FGD, A/F/U Kaduna)

*“If you tell them you do not have money, they dispense drugs to be paid at a later date” (FGD*, Y/F/U Kaduna)

*“The PPMVs are there to serve us any time we knock at their door for services” A/F/R Sanga*

Many of the views expressed by community members, community leaders and patients were corroborated by the PPMVs. 86% of PPMVs reported that there was a health facility within five kms of their outlet, commenting that they were more accessible both geographically and in terms of opening hours. The majority of PPMVs were open 6 or 7 days a week, and over two thirds were open for 13–18 hours a day. Even those with fixed hours were available for clients outside their normal working time.

*“They come to us because we are closer to the community. Secondly, sometimes the problem may occur after close of work so they prefer to come to us and our charges are cheaper.”* (FGD, PPMV)

Two thirds of PPMVs said they were flexible about payment, which they could delay, if requested, but only 16.7% were willing to reconsider the amount charged and most would not accept payment in kind.

### Services provided by PPMVs

Thirty-six PPMVs were interviewed, 75% were male, their mean age was 35 years and 63% had worked as PPMVs for less than 10 years. Six (16.7%) had received formal training in a pharmacy and 72% had been trained through informal apprenticeship. Over half had not received any in-service training, while 17% reported training in the management of specific diseases and 19.4% had been trained in dispensing. Twenty-three percent were nurses or other health professionals, and a third had other occupations, principally farming or hunting.

Observations of 36 PPMV outlets showed that more than half of the outlets provided only medication and medical products and the remainder also sold non-medical products. The outlets had only one room and only half had private space for consultation. 63% had a mains electricity supply and 48% had a refrigerator. 90% of PPMVs kept no medical records but a minority kept a register (15%) or used referral letters (18%).

The vast majority (90%) said they needed more training, in the diagnosis and treatment of illnesses (42%), managing drug supplies (28%) and in new medications (20%).

The commonest complaints PPMVs saw were malaria, fever, back pain, cough and diarrhoea which corroborates some of the findings of the community survey. For all illnesses, PPMVs sold drugs with and without prescriptions, and recommended drugs when necessary. The PPMVs reported that reasons why clients visited PPMVs varied. Some would require specific medication, which could not be supplied by the local hospitals while others would ask PPMVs for advice, but the advice often depended on what the PPMV had in stock. Patients with severe problems were referred to local health facilities.

*“Most people will just come and demand a particular product. If we offer him another, he refuses. Most self-medicate, but some will come and explain the problem before we decide to give them drugs, that is, if I understand his problem. But if I don’t, I refer him.”* (FGD, PPMV)

The commonest drugs sold by PPMVs were analgesics, anti-malarial medication, systemic antibiotics and gentamicin ear drops. Half reported that they administered injections. 50% of outlets sold medication only and others also sold non-medical products. Many PPMVs acknowledged their limitations in providing health care, and those who had a health background were more confident to treat, give injections or even deliver babies.

The PPMVs purchased their drugs from large pharmaceutical stores and from wholesalers in the market, which included suppliers of veterinary drugs. Most reported that the only difficulties they experienced in purchasing drugs was lack of money for the capital outlay. PPMVs reported that they only bought and sold approved medication, although some did not know which drugs were on the approved list. Most PPMVs were familiar with the code pin on packaging introduced by the government to control fake drugs but reported that some manufacturers did not follow the practice. Many mentioned the importance of safe and effective medication.

All PPMVs reported that they occasionally saw clients with eye or ear conditions. The commonest symptoms were discharge or earache, and complaints of vision or hearing loss were uncommon.

*“Yes, some will come with ear problem and demand a particular drop, but I refuse. I will ask further questions to identify the problem before giving the drugs; I feel what is good for the complaint - if it is ear discharge, I give ear drops and if it was dirt that blocked the ear, I clean it up.”* (FGD, PPMV)

Common symptoms of eye conditions were redness, discharge and a feeling of sand in the eyes. Several PPMVs mentioned “Apollo”, the local name given to outbreaks of viral conjunctivitis.

Concerning how they would manage clients with ear or eye problems; for a child with red, discharging eyes or an adult with ear discharge, the PPMVs said that they would usually recommend a medication, or would refer if the condition was severe. When shown an image of a child with purulent discharge in both eyes, 53% thought it was conjunctivitis and 63% would recommend antibiotics. PPMVs were less sure of what was wrong with the woman and the man with declining vision, and 75% would refer the client. They were also unsure of how to manage a painful eye, and a range of treatments and procedures were mentioned, as well as referral to a hospital, which was suggested by 73%.

Many PPMVs did not know what cataracts were or how they are managed, but all would refer clients with “dim vision”. Referral for eye conditions was common, as the eyes were considered “delicate”, as stated by one PPMV:

*“The truth of the matter, the eye is delicate, so we advise them to seek solution at XX hospital. We also advise them against the use of any traditional medicine.” (FDG, PPMV)*

A range of medications were in stock to treat ear conditions, including antibiotics and analgesics, with gentamicin ear drops being the commonest (in 17 outlets). However, around 50% of outlets did not have any medication for ear conditions and 78% of outlets had no ear drops. Findings were very similar for eye medication. Gentamicin and Chloramphenicol eye drops were available in 6 and 1 outlets, respectively, and 78% of outlets had no topical eye medication.

Only 39% of participating PPMVs held a license from the PPMV Association, with the proportion being higher in mixed urban and rural outlets (mixed 70% vs urban only 31% vs rural only 20%). Many PPMVs did not know about the process of obtaining a license but the majority acknowledged the value of having one as it gave them confidence and prevented harassment.

*“There are a lot of advantages [to having a license}, because it protects one from people’s harassment…. We used to have such problems with others - they will come round and collect money with excuses, but once that licence is there, no one will harass and demand… money….One will have confidence because what you are selling, the government are aware of it.”* (FGD, PPMV)

Over 50% of PPMVs had been visited by an official from the PPMV Association. The most reported activities during these visits were to check the environment of the outlet, to remove drugs that were fake or expired, and to caution those selling drugs outside the approved list. Many PPMVs thought the Association could do more in terms of improving their status, training and providing financial assistance.

*“They visited me and checked the drugs I have. They also checked for control and fake drugs which I do not have and they advised me to sell only recommended drugs.”* (FGD, PPMV)

Many PPMVs were willing to be trained in clinical skills and medication specific to eye and ear care.

*“We are all willing to be involved in some training because it will be very important, as none of us has the opportunity to attend any training on ear care.”* (FGD, PPMV)

*“The eye is a delicate organ in life. It is good for one to have more knowledge about it because of the people that come with the problems”. (FDG, PPMV)*

### Training of PMVs and willingness of the community to access PPMVs if they were trained

Despite their negative attitudes to PMVs, most of the health professionals thought they should be trained in ear and eye conditions, focussing on the PMVs who were better educated. Most said they would be willing to be involved in the training.

When participants in the community survey were asked whether they would be willing to go to a PPMV outlet if PPMVs were trained to provide primary ear care services, 41.5% (n = 656/1,581) responded that they would be (35.8% of urban and 47.3% of rural participants). In multivariate analysis the following variables were associated with willingness to visit a PPMV outlet: male sex, no education, no income generating activity, Muslim religion, lived in the community for a long time, and poverty (Table 5). The findings were very similar for eye conditions.

**Table 5:** Factors influencing whether community members would visit a PPMV outlet with an ear/hearing complaint, if the PPMVs were trained to deliver primary ear care.

		Univariate analysis		Multivariate analysis	
Variable		Chi2	p-value	Adjusted odds ratio*	95%CI
Age group	18-29	3.25	0.36		
	30-49				
	50-69				
	70+				
Sex	Male	10.58	0.001	–	–
	Female			0.60	0.47-0.77
Locality	Urban	21.37	<0.001	–	–
	Rural			0.87	0.64-1.17
Education attained	None	31.23	<0.001	–	–
	Any			0.57	0.43-0.76
Income generating activities	None	2.64	0.10	–	–
	Any			0.72	0.53-0.98
Marital status	Single	5.66	0.06	–	
	Married			1.34	0.83-2.21
	No longer married			1.40	0.72-2.73
Religion	Muslim	13.15	0.001	–	–
	Christian			0.64	0.50-0.81
	African traditional/none			0.35	0.03-3.63
Length of stay in community	0-5 years	31.00	<0.001	–	–
	6-20 years			1.82	1.15-2.87
	>20 years			1.90	1.21-2.96
Wealth quintile	First (poorest)	77.10	<0.001	–	–
	Second			0.63	0.45-0.87
	Third			0.57	0.40-0.82
	Fourth			0.28	0.18-0.42
	Fifth (wealthiest)			0.37	0.23-0.58

*Adjusted odds ratio calculated using logistic regression model

Community leaders were positive about the ear and eye care services PPMVs could potentially offer, if they were trained and equipped, as community members expressed a need for services that were local. Although some FGD participants reported that they would be willing to go to PPMVs if they were trained, regulated and properly monitored by competent authorities, opinions were not unanimous.

*“We are ready to accept eye services from the PPMVs if they are properly trained and well stocked with good drugs*”. (FDG Y/F/R Kudan)

*“There is no role for the PPMVs to play in this community, as we all rely mostly on going to the general hospital” (FGD, A/F/R Kudan)*

## Discussion

This study examined community health-seeking behaviour for ear and eye conditions, described the care provided by PPMVs and explored opportunities for the provision of ear and eye care by PPMVs.

As in other studies, our findings show that PPMVs are an important source of health care for many communities in northern Nigeria [11], including for eye care [15]. The findings on community health seeking behaviour are also similar to other studies [18,19] showing that community decision-making processes about seeking care are complex and depend on a number of intrinsic and extrinsic factors. These include socio-demographic factors, the perceived cause and severity of the condition, the availability of financial resources, convenience of access, the efficacy and quality of services, and the attitudes and perceived trustworthiness of providers.

It was evident that community members had to balance what was perceived as a desirable source of care with what was available, accessible, and affordable in reality. For example, government health facilities, which participants preferred for general health conditions, were thought to have appropriately qualified staff and provide a range of diagnostic and treatment services. However, when attending these facilities, many patients were confronted by high consultation fees, inconvenient opening hours, unresponsive staff, inefficient services and shortage of medication. PPMVs on the other hand were seen to be less qualified and unable to provide treatment for more complex conditions, but were readily accessible, had flexible working hours and payment arrangements, and had a range of medications to choose from. These factors likely explain why PPMVs were frequently accessed by participants for general health conditions and why they were not always satisfied with the services they received. Although most participants named formal healthcare providers as their preferred choice of care, in practice, many opted for PPMV services either because PPMVs were more convenient and responsive or because they were the only accessible and affordable option. We have limited information on socio-economic status; however, the data indicate that people in lower socio-economic groups and the uneducated would be more willing to attend PPMVs than wealthier or educated participants. The vast majority of the ear and eye patients interviewed, who were young and urban dwellers, said they had not visited a PPMV prior to attending the hospital. This has been reported in other studies [18,20].

Analysis of PPMV practices show that standards of care varied, and many provided services of sub-optimal quality, such as dispensing the medication requested and at limited doses. Many PPMVs did not have a license and did not know how to obtain one. Many premises lacked appropriate space for patient consultation and facilities to store medication. The fact that many PPMVs did not know which drugs were on the approved PPMV list and mentioned providing a range of services, including injections, suggests that many were practicing outside their legal scope of work with little supervision or control. These findings are in line with other studies of PPMVs in Nigeria. For example, in a study in South West Nigeria, 60–70% of PPMV outlets stocked medication they were legally prohibited from selling [21]. Other studies showed that only 6–20% of PPMVs in Nigeria had a license to practice [22,23] and only 38% were registered with the Pharmaceutical Council of Nigeria [24].

Many PPMVs in our study did not have appropriate training and acknowledged their significant limitations in diagnosis and treatment, a finding reported in our study in relation to eye conditions and in many other studies. For example, in a study which used clinical vignettes, only half of the PPMVs recommended the proper treatment for malaria [20], and in another study, many PPMVs were unaware of oral rehydration therapy for the treatment of diarrhoea [25]. Other studies have reported PPMVs’ poor knowledge of pharmacological and safety guidelines and drug administration [25–27]. However, in a recent study in 16 States across Nigeria, a third of the PPMVs in charge of outlets had some form of medical training, with the proportion being higher in the north than the south. Outlets with trained staff were more likely to stock essential medication for common illnesses, such as artemisinin-based combination therapy for malaria, and to be registered [13].

Although many PPMVs in our study were enthusiastic about providing care for ear and eye conditions, their capacity for treating these conditions was limited. Very few PPMVs, and largely those with some form of medical background, felt confident to treat these conditions but they only had a limited range of appropriate medication. The only option available to the majority of PPMVs was referral of clients to secondary level facilities. However, many PPMV outlets did not keep any medical records and had no means to follow up on referrals.

An important factor which stimulated participants to access PPMV outlets was that health facilities were often out of stock of essential medication, a frequent finding in Nigeria [28]. The administration in Nigeria has recently adopted Universal Health Coverage and has launched **‘**Accelerating progress towards Universal Health Coverage (UHC) through Primary Health Care’ [29]. A new pre-service curriculum has been drawn up for primary health care workers, and resources allocated to improve human resources, infrastructure and maintenance, equipment, essential drugs, vaccines and consumables, with expansion of insurance cover. In addition, the World Health Organization’s Africa region has recently published a training package for primary eye care [30], which has been included in the pre-service curriculum of primary health care workers in Nigeria. Should these initiatives improve primary health care, including for ear and eye conditions, greater confidence in the service may reduce the need for community members to access the services of PPMVs, and primary care could become a point of referral for PPMVs. However, a recent study which used sickness diaries, showed that PPMVs were often visited first, particularly for non-communicable diseases, despite the upgrading of primary care facilities and insurance cover [31]. It is, therefore, likely that PPMVs will remain important in the eyes of communities, and they could play an important role in health ear and eye promotion. To do this they would need better knowledge about the causes and management of the ear and eye conditions prevalent in the local community, and skills in counselling clients. ENT and ophthalmology specialists in our study indicated that they would be willing to be involved in training PPMVs, which would need to be undertaken in collaboration with the PPMV Association and other relevant authorities, and with the approval of community leaders to build trust. A pilot study would be needed in the first instance to assess the impact of training on PPMVs’ practices, both harmful and beneficial.

There were several limitations in the study. Individuals interviewed in the hospital outpatient clinics may have been reluctant to admit that they had previously visited a PPMV or other informal providers of health care, thinking that it might compromise their care in the hospital. Few community members had an ear or eye condition at the time of the survey, but in the FDGs they ranked these conditions highly. This may reflect acquiescence bias, as the purpose of the study was described in the information sheet used to obtain consent. The sample of PPMVs was also too small to compare their level of training with their knowledge of ear and eye conditions and how they would manage them. Lastly, in-depth interviews may have been more appropriate than FGDs for the hospital staff providing ENT and ophthalmic services.

Our findings across all data sources consistently suggest that PPMVs are likely to remain an important source of care for a significant proportion of the Nigerian population, particularly those with limited resources and poor access to formal healthcare providers. Some limitations in the general care they provide were identified in this study, which suggests that improving PPMVs’ knowledge and skills should take place alongside other systemic changes, including mechanisms for regular supervision, effective procurement and supply chain management, and improved information systems.

### Conclusions

Alongside their usual practice, PPMVs could play an important role in primary ear and eye health care in the community, particularly in health promotion and advice about where to seek formal care. This would require training on the symptoms and signs of common eye and ear problems, their prevention and detection and when to refer clients. Training in counselling would also be useful, to enable them to counsel clients to alleviate anxiety, counter misunderstandings, and encourage uptake of referral, if required. This task shifting would need to fall within the regulatory framework for PPMVs, and the curriculum and training would need to be developed and delivered as a collaboration between the PPMV Association and local clinicians. However, training such a large number of people and ensuring they provide good quality care, with feedback on referrals and supportive supervision, would pose considerable challenges.

## Abbreviations

PPMV: Proprietary and patent medicine vendors
WHO: World Health Organization
